# Ewing's sarcoma of the head and neck

**DOI:** 10.1590/S1516-31802000000600010

**Published:** 2000-11-01

**Authors:** Adriano Santana Fonseca, Raquel Mezzalira, Agrício Nubiato Crespo, Antônio Emílio Bortoleto, Jorge Rizzato Paschoal

**Keywords:** Sarcoma, Ewing's, Pharynx, Mandible, Head and Neck Surgery, Otolaryngology, Sarcoma de Ewing, Mandíbula, Cirurgia de Cabeça e Pescoço, Otorrinolaringologia

## Abstract

**CONTEXT:**

Ewing's sarcoma is a rare neoplasm, which usually arises in long bones of the limbs and in flat bones of the pelvis, with the involvement of head and neck bones being very unusual.

**CASE REPORT:**

a case of Ewing's sarcoma occurring in the mandible of a 35-year-old female. Pain and swelling of the tumor were the main complaints. The early hypothesis was an undifferentiated malignant neoplasm, possibly a sarcoma. The CT scan depicted an expansive lesion, encapsulated, with septa and characteristics of soft tissue, involving the left side of the mandible and extending to the surrounding tissues. The patient underwent surgical excision of the lesion, the definitive diagnosis of Ewing's sarcoma was established, and the patient commenced on radiotherapy.

## RESUMO

**CONTEXTO:**

O Sarcoma de Ewing é uma doença que geralmente acomete os ossos longos e a pelve, sendo rara a ocorrência em cabeça e pescoço.

**RELATO DE CASO:**

Caso de Sarcoma de Ewing começando na mandíbula em uma paciente de 35 anos, sendo a tumoração e a dor local os principais sintomas referidos. O diagnóstico inicial foi de neoplasia maligna indiferenciada, sugestivo de sarcoma e a tomografia computadorizada mostrou lesão expansiva com características de tumor de partes moles, septada e encapsulada, acometendo a mandíbula esquerda e apresentando extensão para as partes moles adjacentes. O tratamento realizado foi a remoção cirúrgica da lesão, complementado com radioterapia pós-operatória. O diagnóstico definitivo foi de Sarcoma de Ewing.

## INTRODUCTION

Ewing's sarcoma is an uncommon malignant neoplasm, locally aggressive, which occurs more often in males than females, and in the first three decades of life.^1^ Long bones are the most common site. The tumor is very rare in otolaryngology practice, but it may be found involving the mandible, cervical vertebrae and the temporal bones.

## CASE REPORT

A thirty-five-year-old Caucasian female presented swelling on the left side of the mandible, in August 1994, which had been present for seven months. She also complained of painful nodulations in the surrounding gums, which were compromising her mastication and speech, and causing otalgia and odontalgia. Fifteen years earlier she had had her teeth extracted and she was using orthodontic prostheses on the lower and upper arcades.

On examination a firm painful lesion involving the angle and body of the mandible, as well as the left submandibular region and the floor of the oral cavity, was noted. Paresthesia of the left lower arcade was also identified.

**Figure 1:**
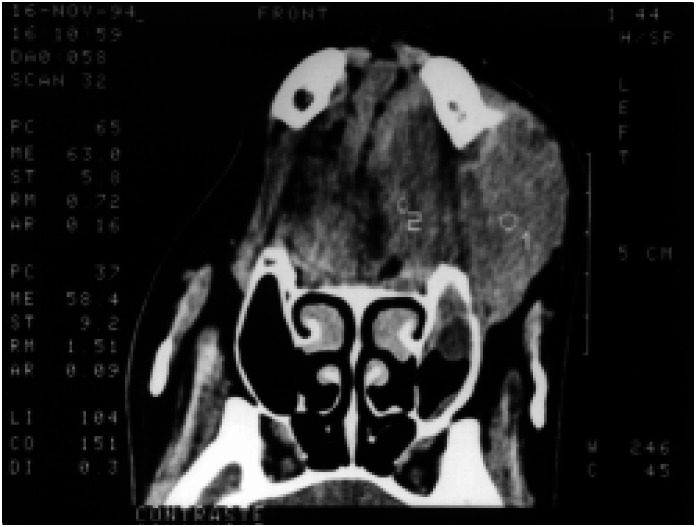
CT scan showing an expansive lesion on the left portion of the mandible, causing deviation of the tongue away from the midline.

**Figure 2:**
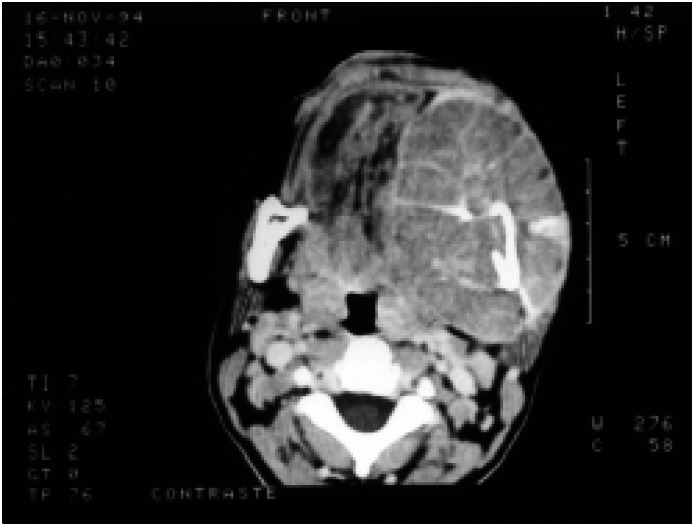
CT scan of a capsulated mass involving the mandible with septa and deviation of the surrounding tissues.

The biopsy showed a pattern suggestive of undifferentiated malignant neoplasm. The immunohistochemical stains pointed the diagnosis towards a sarcoma. The CT scan depicted an expansive lesion with opacity, compatible with soft tissues, capsulated and with septa, involving the left portion of the mandible and extending to the surrounding tissues *(Figures 1 and 2)*.

The patient underwent left hemimandibulectomy and tracheostomy in the same surgical procedure. The histological findings showed a small round cell malignant neoplasm of the bone, compatible with Ewing's sarcoma with neoplastic invasion of the adjacent soft tissues. The surgical margins were not compromised.

Afterwards, the patient was treated with radiotherapy. The cervical region and then the left side of the face were treated to a total dose of 50 Grays, in twenty-five exposures of two Grays. The patient has been in follow-up for four years and is free of disease and feeling well.

## DISCUSSION

Ewing's sarcoma is an unusual disease comprising about 4 to 6% of all primary bone tumors. It originates in the marrow cavity and is found in the epiphyses of long and flat bones. Involvement of the head and neck in Ewing's Sarcoma is very unusual, accounting for approximately 1 to 4% of cases. The skull and mandible are the most frequent sites.^1,2^ It is rare after the third decade and occurs most often in the second decade. It is more common in males than females.^3^

Generally it presents with pain and local swelling, dilated veins, hyperthermia, anemia, increased erythrocyte sedimentation rate and leukocytosis.^2,3,4^ A history of previous trauma is present in many reported cases. The initial evaluation includes radiographic study of the suspected area, which shows areas of bone rarefaction, frequently associated with increased density, periosteal reaction, and bone neoformation resulting in an "onion layers" appearance.^3^

The diagnosis is established by biopsy, in which the tumor is seen as layers of small round cells, similar to lymphocytes, but larger. Mitotic cells are rare, intercellular stroma is scarce and a large portion of the tumor may be necrotic. Tumor cells placed around a clear central area forming rosettes may be seen, resembling Homer-Wrigt rosettes, typical of neuroblastomas. Intracytoplasmic glycogen is a definitive aspect, but not pathognomonic because it is also present in other primitive tumor cells such as osteosarcomas, rhabdomyosarcomas and neuroblastomas.^3,4^ Hence, the differential diagnosis of Ewing's sarcoma encompasses a wide number of diseases including osteosarcomas, rhabdomyosarcomas, neuroblastomas,

mesenchymal chondrosarcoma and malignant lymphoma.

The appropriate treatment for Ewing's sarcoma has been the surgical excision of the tumor associated with radiotherapy and chemotherapy.

Due to the high local reoccurrence rate (20%) following radiation therapy alone, radical surgical removal must be attempted to increase local control whenever feasible. The same applies to the mandible, since today's reconstruction techniques alleviate esthetical and functional impairment to the patient. Radiotherapy must be used as neoadjuvant therapy or in non-resectable primary radiosensitive tumors. Chemotherapy must be reserved to prevention and treatment of metastasis.

The association of surgery, radiotherapy and chemotherapy has significantly improved the 5-year survival ratio, now reaching 40 to 75%.^3^ The single most important indicator is the primary site, and thus, primary tumors of the head and neck, especially the mandible, have a significantly higher survival ratio.^2^ An extremely high erythrocyte sedimentation rate has been associated with a poorer prognosis. Death is usually due to disseminated hematogenic spreading of the disease.
